# MKNet-family architectures for auto-segmentation of the residual pancreas after pancreatic resection: a deep learning comparative study

**DOI:** 10.1007/s00261-025-05211-4

**Published:** 2025-11-27

**Authors:** Dennis Böhm, Paul C. M. Andel, Paul A. Akkermans, Bas Boekestijn, Willem van der Geest, Robbert J. de Haas, Jakob W. Kist, I. Quintus Molenaar, Joost Nederend, C. Yung Nio, Bobby K. Pranger, Hjalmar C. van Santvoort, Femke Struik, Inez M. Verpalen, Frank J. Wessels, Wouter B. Veldhuis, Helena M. Verkooijen, François E. J. A. Willemssen, Ralf I. Zoetekouw, Jouke Dijkstra, Martijn P. W. Intven, Michael Weinmann, Lois A. Daamen

**Affiliations:** 1 Datacation B.V., Eindhoven, The Netherlands; 2 Delft University of Technology, TU Delft, The Netherlands; 3 Regional Academic Cancer Center Utrecht, UMC Utrecht Cancer Center & St. Antonius Hospital Nieuwegein, Department of Surgery, Utrecht, The Netherlands; 4 Medical Spectrum Twente, Department of Radiology, Enschede, The Netherlands; 5 Leiden University Medical Center, Department of Radiology, Leiden, The Netherlands; 6 University Medical Center Groningen, Department of Radiology, Groningen, The Netherlands; 7 Amsterdam UMC, location University of Amsterdam, Department of Radiology, Amsterdam, The Netherlands; 8 Catharina Hospital Eindhoven, Department of Radiology, Eindhoven, The Netherlands; 9 UMC Utrecht Cancer Center, Department of Radiology, Utrecht, The Netherlands; 10 University Medical Center Utrecht, Division of Imaging and Oncology, Utrecht,, The Netherlands; 11 Erasmus MC, University Medical Center Rotterdam, Department of Radiology and Nuclear Medicine, Delft, The Netherlands; 12 UMC Utrecht Cancer Center, Department of Radiation Oncology, Utrecht, The Netherlands

**Keywords:** Pancreas, Surgery, CT scan, Segmentation, Deep learning

## Abstract

**Purpose:**

Accurate interpretation of CT scans after pancreatic resection is crucial for detecting abnormalities, including postoperative complications and cancer recurrence. This study investigates the feasibility and clinical utility of a novel MKNet-family deep learning architecture for auto-segmentation of the residual pancreas on postoperative CT imaging, in comparison to previous approaches.

**Method:**

Novel MKNet, MSKNet and MAKNet architectures were developed. Two datasets were used: the National Institutes of Health (NIH) dataset, comprising 82 annotated normal preoperative CT scans, and the IMPACT Consortium dataset (NCT06055010; https://github.com/IMPACTconsortium/IMPACT), comprising 81 annotated postoperative CT scans obtained < 4 weeks after pancreatectomy. Performance was assessed by Hausdorff Distance (HD), 95th-percentile-HD (HD95) and Normalized Surface Distance (NSD), and secondarily by Dice Similarity Coefficient (DSC), and compared with self-implemented existing models for preoperative pancreas auto-segmentation. Qualitative evaluation was conducted by ten abdominal radiologists.

**Results:**

In the postoperative setting, the MAKNet architecture showed the best performance, with an HD and HD95 of 17.3 ± 11.2 mm and 11.5 ± 10.2 mm, respectively. DSC (64.9 ± 14.8%) and NSD (27.2 ± 8.2%) were comparable to the Attention-U-Net (DSC 66.0 ± 13.8%; NSD 27.8 ± 8.4%). Clinical evaluation indicated that the MKNet-family accurately defined the postoperative pancreas (i.e., requiring minimal or no modifications) in 64 of 81 segmentations (79%).

**Conclusion:**

This study demonstrates the effectiveness of novel MKNet-family architectures to accurately segment the residual pancreas on postoperative CT imaging over previous approaches. This advances the state-of-the-art in pancreas auto-segmentation and may be beneficial for medical application and education, acceleration of data annotation, and future research.

**Supplementary Information:**

The online version contains supplementary material available at 10.1007/s00261-025-05211-4.

## Introduction

Surgery is commonly performed to treat (pre)malignant diseases of the pancreas and biliary tract [1]. Pancreatoduodenectomy is the most frequently performed pancreatic surgery, involving removal of the pancreatic head, duodenum, gallbladder and bile duct, with subsequent reconstruction of the gastrointestinal tract by creating new anastomoses between the partially removed organs [2–4]. This complex procedure is associated with a substantial risk of developing postoperative complications such as fluid collections, abscesses and anastomotic leaks, along with the development of dense postoperative fibrosis. These changes, in addition to the existing variation in size, shape, and location of the pancreas between individuals, obscure the anatomical landmarks and create additional challenges for accurate segmentation of the pancreatic remnant on postoperative CT scans [5, 6]. This might demand expertise that is not always available. Accurate segmentation of the pancreatic remnant on CT scans after pancreatic resection, however, is important to distinguish it from abnormalities, including well-known issues such as postoperative complications and cancer recurrence, to enable prompt initiation of treatment if needed [7, 8].

Recent developments in data science, notably the emergence of artificial intelligence (AI) and Deep Learning (DL), have shown promise in enhancing several aspects of medical imaging analysis such as image segmentation [9–12]. An algorithm capable of accurately auto-segmenting the pancreatic remnant could enhance further imaging analysis, including automated lesion detection and characterization, and radiomics analysis. This has the potential to contribute to further optimization of workflow processes, reduction of clinician workload and minimization of interobserver variability regarding the characterization of findings in the pancreatic region. Furthermore, it can offer valuable support for educating and guiding less experienced radiologists and expedite labor-intensive manual annotation of research data, thus providing a promising tool for research, education and clinical practice [5, 6].

Previous studies have focused on pancreas segmentation in the preoperative setting which is not complicated by the extensive changes that are present after pancreatic resection [9, 13–29]. Training and validation of preoperative pancreas auto-segmentation models have predominantly relied on the publicly available pancreatic CT dataset from the National Institutes of Health (NIH) [30], with current state-of-the-art algorithms achieving Dice Similarity Coefficient (DSC) scores up to 88% [16, 17]. However, it is likely that these algorithms may demonstrate suboptimal performance in the postoperative context, considering the even wider variation of organ structures between patients, and require further optimization. Simple convolutions such as in a basic U-Net are considered insufficiently effective to grasp the complex characteristics of the pancreas in the postoperative setting [16, 17]. Therefore, a model with a high degree of flexibility and shape awareness is warranted. No prior studies have been conducted on automated postoperative pancreas segmentation. This study investigates the feasibility and clinical utility of a novel MKNet-family deep learning architecture, specifically developed for auto-segmentation of the residual pancreas on postoperative CT imaging, in comparison to previous auto-segmentation approaches used in the preoperative setting.

## Methods

This study was reported according to the Checklist for Artificial Intelligence in Medical Imaging (CLAIM) [31].

### Study design

A retrospective study was performed to create a model for auto-segmentation of the residual pancreas after pancreatic resection, using an experimental and iterative approach. The institutional board approved this study (UMC Utrecht register no. 212191910), and confirmed that it does not fall under the scope of the Dutch Medical Research Involving Human Subjects Act (WMO).

## Image datasets

Two datasets were used for training and validation (Table 1): (1) a publicly available dataset with pancreatic CT scans from The Cancer Imaging Archive (TCIA) created by the NIH [30]; and (2) a dataset from the IMPACT Consortium (NCT06055010; https://github.com/IMPACTconsortium/IMPACT). The NIH dataset consists of 82 contrast-enhanced CT scans of healthy individuals, e.g., without pancreatic abnormalities, in the porto-venous phase [30]. In this dataset, the pancreas was manually segmented slice-by-slice by a medical student and verified or modified by an experienced radiologist using UMC Utrecht Volumetool software. The IMPACT dataset contained 81 contrast-enhanced abdominal CT scans in the porto-venous phase, in accordance with our institution’s CT pancreas protocol. Mean number of slices per CT scan was 204 (range 86–467). Scans were performed in patients who underwent partial pancreatic resection for either benign or (pre)malignant indications. Postoperative CT scans were performed in case of suspected complications or as part of the postoperative follow-up. On each porto-venous CT scan, the residual pancreas was annotated per slice by a postdoctoral researcher, radiology resident or experienced radiation oncologist, the latter of whom verified all annotations.

**Table 1:** Specifications of the imaging datasets used for training and validation

	NIH dataset (*N* = 82) [30]	RACU dataset (*N* = 81)
**Setting**	Preoperative	Postoperative
**Phase**	Porto-venous phase, ~ 70 s after intravenous contrast injection	Porto-venous phase, ~ 55 s after intravenous contrast injection (according to the CT pancreas protocol)
**Resolution in pixels**	512 × 512	512 × 512
**Number of slices**	Various	Various
**Pixel sizes**	Various	Various
**Slice thickness in mm**	1.5–2.5	0.8–4
**Manufacturer**	Philips and Siemens	Philips and Siemens
**Scanner type**	MDCT	MDCT
**Tube voltage in kVp**	120	100 or 120
**Annotations**	Medical student, verified/modified by experienced radiologist	Two postdocs (MD) and one radiology resident or experienced abdominal radiation oncologist, the latter of whom verified all annotations

## AI framework development

### Data preprocessing

All CT scans were acquired in DICOM format and converted to NIFTI files to ease processing using the PlatiPy Python library [32]. Intensity values were clipped between [−100, 240], corresponding to a window width of 340 and window level of 70. During training, the input intensities were scaled between 0 and 1 using Min-Max scaling to enhance convergence and prevent gradient overflow. Since the data was from different CT scanners and had different slice thicknesses, the voxels were resized to be 1 mm x 1 mm x 1 mm using trilinear interpolation for the CT images and nearest neighbors for the binary masks, following the approach of Salantri et al. [17] During each training cycle, a random sample of 128 × 128 × 64 voxels was taken from each CT scan with a probability of 50% that the center voxel is a pancreatic voxel. This was done to reduce memory impact.

## Model architecture

**Fig. 1:**
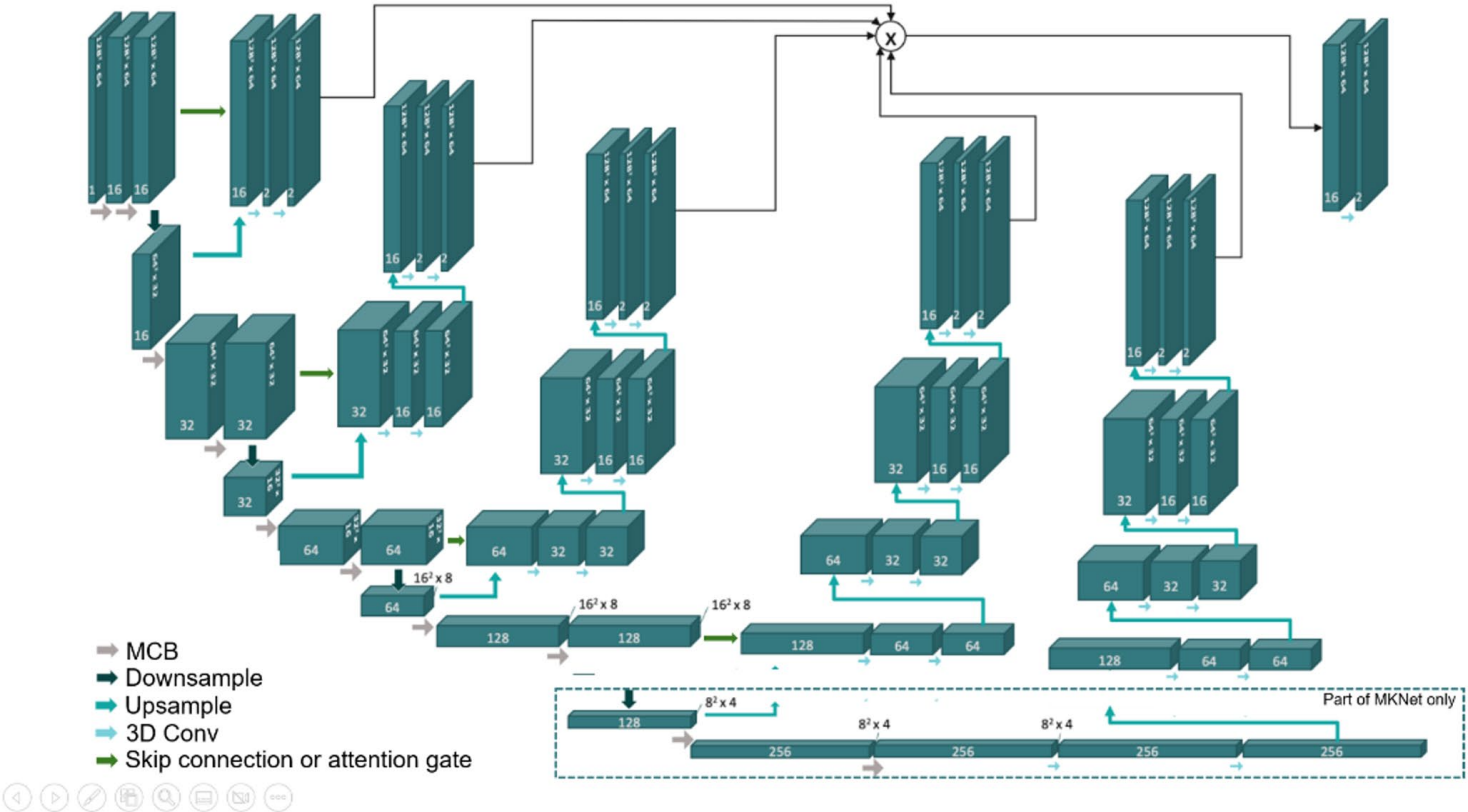
The novel encoder-decoder Multi-scale KNet (MKNet) family architectures based on elements of PanKNet and MHSU-Net [15, 16]

Model development was inspired by the beneficial impact of using Multi-Scale Convolutional Blocks (MCBs) within previously developed MHSU-Net [16] and PanKNet [17] (Supplementary Table 1). These architectures are based on the idea that simple convolutions in a basic U-Net are not sufficient to grasp the complex characteristics of the pancreas [16, 17]. During pancreatic resection, different parts of the pancreas and surrounding organs are removed, depending on the location and extent of the pancreatic lesion. This requires the model to have an even higher degree of flexibility and shape awareness in the postoperative setting. To achieve this, we developed a novel encoder-decoder Multi-scale KNet (MKNet) architecture (Fig. 1). The encoder uses newly designed 3D MCBs, consisting of three branches with one, two and three consecutive convolutions, respectively. Similar to the KNet architecture, our MKNet has multiple decoders for each layer that outputs a segmentation mask. All masks are concatenated in the channel dimension and reduced using a convolution to get to a single segmentation. Potential beneficial elements of U-Net architectures are skip connections, directly transferring information from different encoder layers to their decoder counterpart and, thereby, supporting the preservation of high-frequency characteristics in the result [20, 21]. As the layer-specific decoders of our MKNet should already contain information from corresponding encoders, skip connections would be redundant. To test this hypothesis, a second MKNet with skip connections was created: the Multi-scale Skip connection KNet (MSKNet) (Fig. 1). The addition of skip connections also allowed addition of attention mechanisms similar to the one used in the Attention U-Net [22], resulting in a third MKNet variant: the Multi-scale Attention KNet (MAKNet) (Fig. 1).

MKNet-family models were pre-trained on the preoperative data from the NIH dataset, after which the best performing instance of each model was further trained and fine-tuned on the postoperative data. The architectures were trained using 4-fold cross validation [13–17, 19, 33]. All MKNet-family architectures were trained using the Novograd optimizer with an initial learning rate of 0.001, a batch size of 8. No maximum number of epochs was defined. Early stopping was applied based on the validation DSC. Considering that the models were trained on random 128 × 128 × 64 samples of the original volume, batch normalization is not representative and layer normalization was applied. In combination with layer normalization, Mish activation [34] showed the best performance. Experiments showed that a kernel size of 3 × 3 × 3 within the MCBs provided the best balance between contextual information and generalization. To address data imbalance resulting from a substantially larger fraction of background voxels in comparison to pancreatic voxels, the loss function used for training existed of a combination of Dice and Focal Loss [35–37]. For down-sampling, the MaxAvg module outperformed the convolutional pooling [36] module and was therefore used.

### Model evaluation

Model performance was assessed both in the preoperative setting and postoperative setting. Performance was compared to different state-of-the-art algorithms (TotalSegmentator [9], PanKNet [17], PanKNet_Light_ [17], and a self-implemented version of MU-Net [16]) and commonly used U-Net [20, 21] and Attention U-Net [22] implementations of MONAI [39].

TotalSegmentator (v2.10.0), which is based on the nnU-Net framework, encompasses one of the most widely used, state-of-the-art multi-organ segmentation models and was therefore used to establish a reference standard and to quantify domain shift [9, 29]. In line with its intended use, it was applied as published, in a zero-shot setting (i.e., without any further fine-tuning or training).

For the experiments of UNet and AttentionUNet, the MONAI framework was used for implementation. Hyperparameters were taken from original papers when provided. For PanKNet_Light_ and PanKNet, the exact same implementation as published by the authors was used, using their hyperparameters. No code was available for the MUNet, meaning we created a new implementation using the UNet implementation of MONAI as a basis, and changing the proposed implementation to a 3D model. Preprocessing steps were harmonized across all models and identical to these used for the MKNet-family models, unless the original code of a baseline model required a different approach. Specifically, all images were cropped to [−100, 240], normalized to [0, 1], resampled to 1 mm x 1 mm x 1 mm voxel spacing, and random crops of 128 × 128 × 64 voxels were used per scan per epoch. For PanKNet, the preprocessing from the official code was followed. After pretraining, all baseline models except for TotalSegmentator underwent the same training and fine-tuning procedures on the postoperative data as the MKNet-family models.

The average DSC (%), Hausdorff Distance (HD; mm), 95th -percentile HD (HD95; mm), and Normalized Surface Distance (NSD; %) of the 4-fold cross validation were calculated and presented as mean ± standard deviation (SD). DSC only focuses on pixel-wise classification accuracy, while HD and HD95 provide shape-aware evaluations, and NSD provides boundary aware-evaluations. For this particular task, HD, HD95 and NSD were therefore considered most relevant. MONAI [39] was used for computation of DSC, HD and HD95; DeepMind’s *Surface distance metrics* implementation [40] was used for NSD. NSD threshold values were arbitrarily set to 3 mm for the X- and Y-axes and 2 mm for the Z-axis. For HD and HD95, a lower score represents a better performance, whilst this is reflected by a higher score for DSC and NSD. Sensitivity analysis was conducted to show performance after stratification for resections of the pancreatic head (i.e., pancreatoduodenectomy) or tail (i.e., distal pancreatectomy).

In addition to quantitative metrics, the novel MKNet architecture was qualitatively evaluated by ten independent abdominal radiologists who each carefully analyzed all model segmentations. The radiologists received all pancreatic slices in which the circumference of the segmentation was highlighted, with a 10 mm margin in cranio-caudal direction. For each case, they were asked to classify the outcomes into: (1) No adjustments; (2) Minor adjustments; (3) Substantial adjustments; and (4) Major adjustments. Subsequently, they were asked on a 5-point Likert scale how likely they would use the model again for pre-annotations: very unlikely, unlikely, neutral, likely, or very likely. The Fleiss’ kappa coefficient with corresponding 95% confidence interval (CI) was computed to assess agreement between the radiologists. The final classification was assessed by calculating the modus, i.e., the most frequently occurring classification in the dataset.

## Results

### Participant demographics

The NIH dataset included 82 CT scans of 53 male and 27 female patients, of whom 17 patients were healthy kidney donors scanned prior to nephrectomy [29]. The remaining 65 patients had neither major abdominal pathologies nor pancreatic lesions. The mean age was 47 (SD ± 17; range 18–76) years. Mean volume of pancreatic tissue in the NIH dataset was 73,510 mm^3^. The IMPACT dataset included 81 scans of 42 male and 39 female patients. Fifteen patients underwent pancreatic resection for a benign indication, whilst 66 patients had a pancreatic malignancy. Pancreatic resection consisted of pancreatoduodenectomy in 61 patients and distal pancreatectomy in 20 patients. Mean age was 67 (SD ± 11; range 29–84) years (Table 1). Mean volume of pancreatic tissue in the IMPACT dataset was 23,290 mm^3^.

### Quantitative performance

Re-implementation of the models described by Ma et al. [16] and Salanitri et al. [17] in the NIH dataset did not allow reproduction of the DSCs reported in their papers, with a decrease in performance ranging from 7 to 12% (Table 2). In the preoperative setting, the MKNet achieved the best performance regarding the HD and HD95, which were 13.6 ± 5.4 mm and 5.9 ± 3.6 mm, respectively. The Attention U-Net had the highest DSC and NSD of 83.1 ± 4.9% and 42.7 ± 6.5%, respectively.

**Fig. 2:**
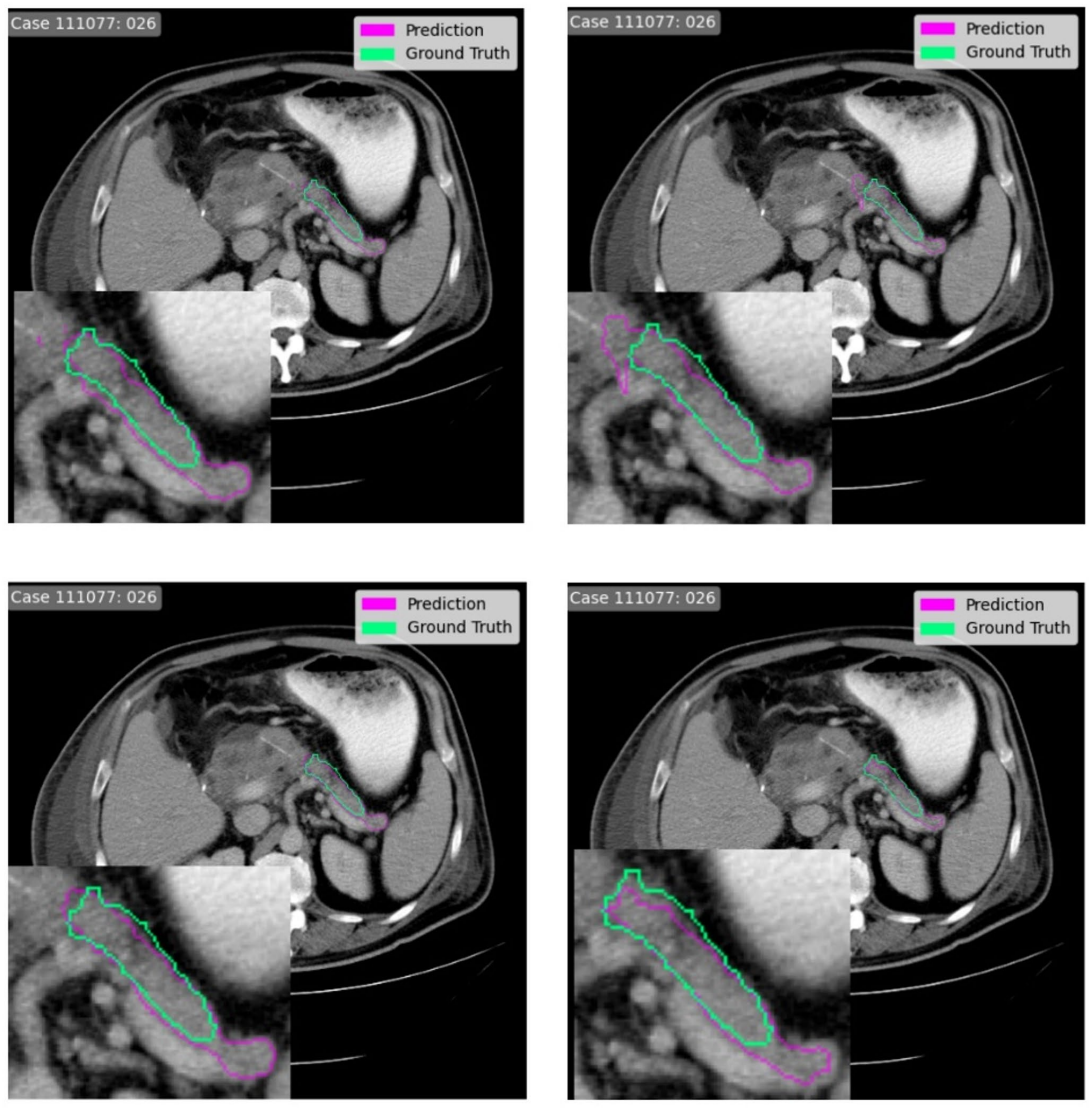
Segmentations of the MKNet-family compared to the current state-of-the-art Attention U-Net for case 111,077

All models performed worse in the postoperative setting, with a ± 20% decrease in DSC, a 3–7 mm higher HD, almost doubled HD95, and a ± 15% decrease in NSD (Table 2). Best performance with regard to HD (17.3 ± 11.2 mm) and HD95 (11.5 ± 10.2 mm) was achieved with the MAKNet. The Attention U-Net showed again the highest DSC (66.0% ± 13.8 mm) and NSD (27.8% ± 8.4 mm). An example of a segmentation by the MKNet-family and the Attention U-Net is visualized in Fig. 2, as compared with the ground truth, representing one slice of a subject who underwent pancreatoduodenectomy. Stratified analysis after finetuning for pancreatic head or tail resections is shown in Supplementary Table 2.

**Table 2:** Quantitative performance of the novel MKNet-family architecture, as compared to implemented state-of-the-art algorithms for pancreas auto-segmentation

Model	Preoperative setting					Postoperative setting			
	Reported* DSC (%), mean ± SD	Achieved* DSC (%), mean ± SD	HD (mm) mean ± SD	HD95 (mm) mean ± SD	NSD (%), mean ± SD	DSC (%), mean ± SD	HD (mm), mean ± SD	HD95 (mm), mean ± SD	NSD (%), mean ± SD
**TotalSegmentator** [9, 29]^**#**^	70.0 ± NR	80.7 ± 10.7	18.1 ± 12.1	8.7 ± 8.8	23.0 ± 6.3	61.2 ± 14.4	47.3 ± 26.7	31.2 ± 23.7	25.2 ± 11.0
**U-Net** [19, 21]^**#**^	82.0 ± 4.3	82.1 ± 5.5	15.5 ± 8.1	6.9 ± 5.7	40.5 ± 6.9	63.9 ± 16.1	21.1 ± 15.0	14.9 ± 13.0	26.4 ± 8.7
**Attention U-Net** [22]	83.1 ± 3.8	**83.1 ± 4.9**	15.7 ± 9.1	7.1 ± 7.1	**42.7 ± 6.5**	**66.0 ± 13.8**	19.7 ± 13.9	13.3 ± 12.7	**27.8 ± 8.4**
**PanKNet** [17]	88.0 ± 4.7	79.8 ± 7.6	16.6 ± 11.9	8.5 ± 10.1	34.8 ± 6.2	62.9 ± 15.9	22.1 ± 17.4	16.1 ± 16.5	23.6 ± 7.4
**PanKNet**_**Light**_ [17]	87.1 ± 4.6	76.9 ± 7.0	18.3 ± 11.1	9.6 ± 8.9	29.1 ± 5.3	59.7 ± 15.3	20.4 ± 12.8	14.3 ± 11.9	20.8 ± 7.1
**MU-Net** [16]	**88.1 ± NR**	81.8 ± 7.1	16.9 ± 12.5	7.9 ± 10.1	40.9 ± 7.2	61.9 ± 16.8	22.3 ± 17.0	16.1 ± 15.1	26.0 ± 8.9
**MKNet (ours)**	NA	81.5 ± 6.4	**13.6 ± 5.4**	**5.9 ± 3.6**	39.2 ± 7.2	62.5 ± 14.6	20.2 ± 12.2	14.1 ± 11.4	25.8 ± 9.5
**MSKNet (ours)**	NA	82.2 ± 6.1	15.1 ± 8.6	6.7 ± 6.6	41.3 ± 6.9	64.4 ± 14.6	18.8 ± 11.8	12.7 ± 11.1	27.5 ± 8.1
**MAKNet (ours)**	NA	81.6 ± 6.7	17.0 ± 10.4	8.0 ± 8.2	40.9 ± 7.6	64.9 ± 14.8	**17.3 ± 11.2**	**11.5 ± 10.2**	27.2 ± 8.2

*DSC* Dice Similarity Coefficient, *SD* Standard deviation, *HD* Hausdorff Distance, *HD95* 95th -percentile Hausdorff Distance, *NSD* Normalized Surface Distance, *NR* Not reported *NA* Not applicable

*Reported DSC values are based on literature. Achieved DSC values are based on our implementation. Note: Reported values are drawn from the original studies. Test splits and evaluation setups may differ from each other and those used in the current study; comparisons are approximate

^#^TotalSegmentator (Version 2.10.0) [29] was applied zero-shot and used as reference standard; comparisons are approximate

^$^Many different scores have been reported for the U-Net in segmentation of the pancreas, varying from 71.8% in the original U-Net paper up until 87.6%. These differences can be partly explained by different data processing methods and minor changes in the network architecture. To give a representative number that seems to be close to the median, the score reported by Oktay et al. [22] was used

### Qualitative performance

**Fig. 3:**
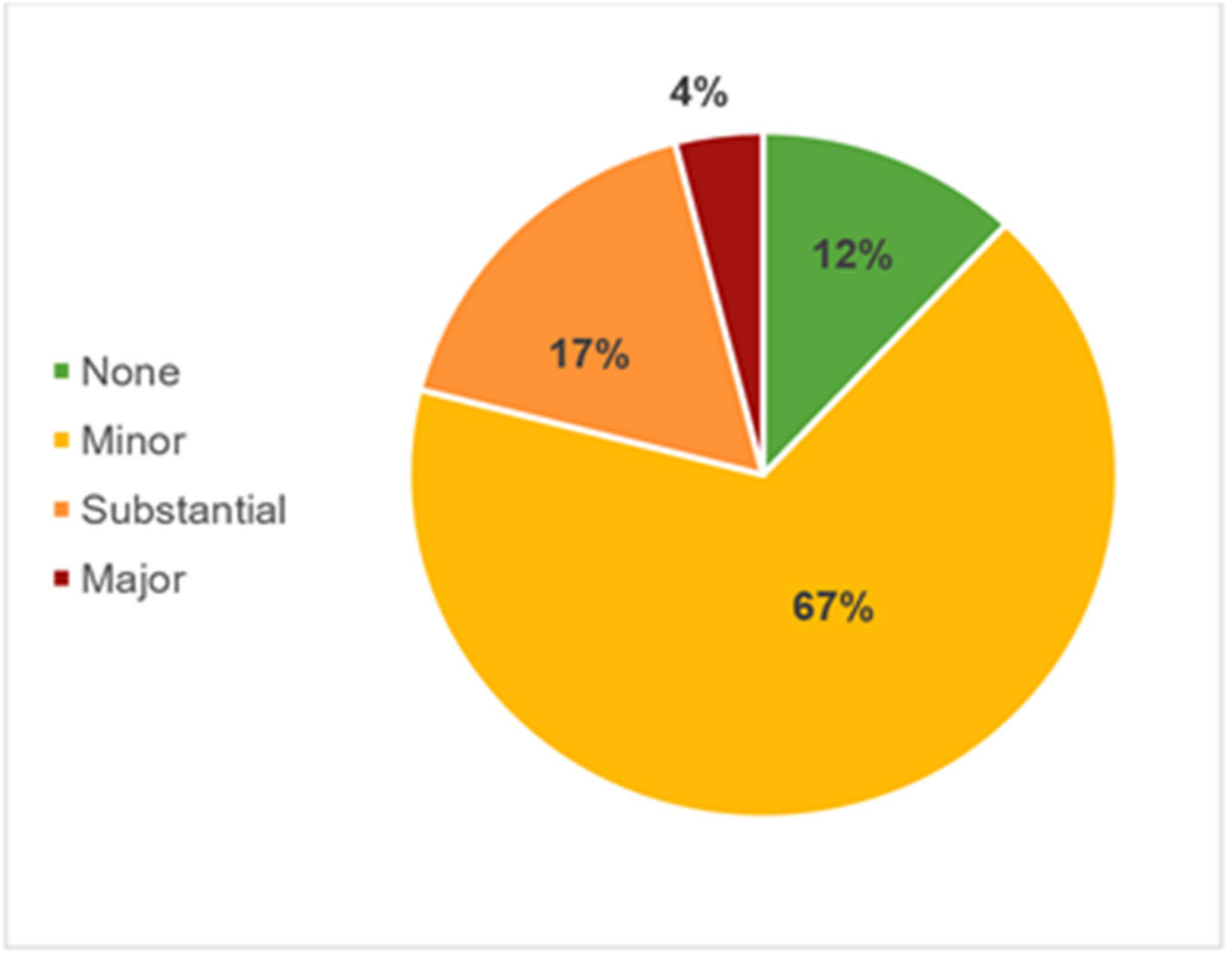
Pie-chart demonstrating whether adjustment was necessary for clinical utility of the MKNet architecture for postoperative pancreas segmentation

MSKNet segmentations were used for qualitative evaluation. Visual examples of single slices of the postoperative pancreas segmentation by the MSKNet model are shown in Supplementary Fig. 1. Expert radiologists indicated that 12% and 67% of segmentations required no or minor adjustments, respectively (Fig. 3). Substantial adjustments were needed in 17%, and major adjustments in 4% of segmentations. Eight experts stated that they would likely (*n* = 6) or very likely (*n* = 2) use the algorithm for pre-annotations. Two experts indicated that they were neutral (*n* = 1) or unlikely (*n* = 1) to use the algorithm again. The radiologist who was neutral specifically mentioned that it would be useful for less experienced clinicians.

Only one case was unanimously classified into the same category by all radiologists. Seven of ten radiologists assigned the segmentation to the same category for 30/81 of the segmentations (37%). In 30 cases (37%), segmentations were classified as ‘no adjustments necessary’ by at least one radiologist, whereas at least one other radiologist classified it as ‘substantial adjustments necessary’. Fleiss’ kappa score was 0.23 (95% CI 0.20–0.25, *p* < 0.001).

**Fig. 4:**
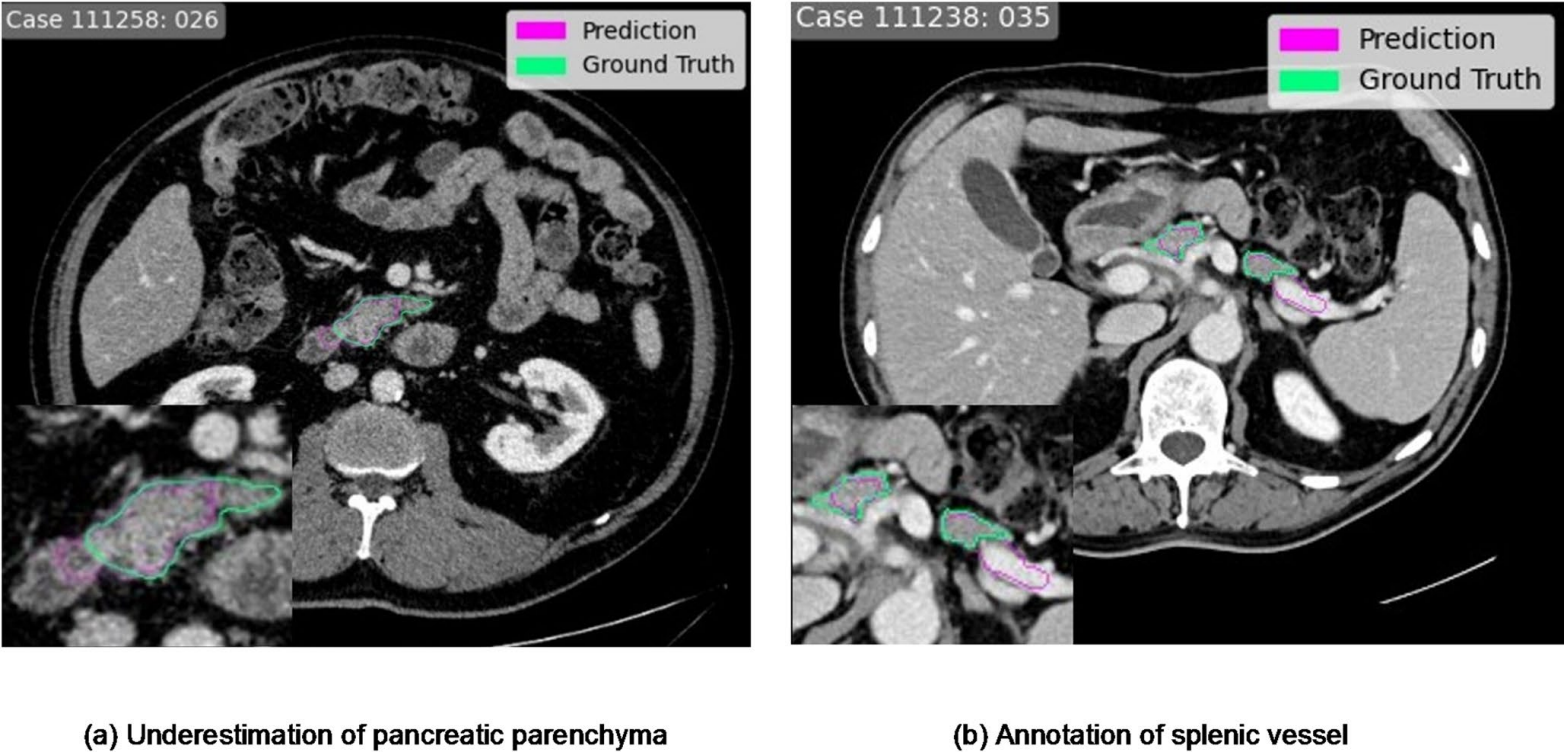
Segmentation of the MKNet-family illustrating **a** underestimation of the pancreatic parenchyma, and **b** annotation of splenic vessels

Further qualitative analysis of the algorithmic performance by the expert radiologists revealed that the algorithm tends to underestimate the pancreatic parenchyma (functional pancreatic tissue, ed.), which worsens when there is substantial atrophy, fatty infiltration, or cystic areas. Hypodense regions were not properly included, which was particularly evident in the pancreatic head. At the level of the head, the algorithm was considered to show difficulties to differentiate pancreatic tissue from the duodenum, often resulting in an underestimation (Fig. 4a). It performed better in the tail, although the segmentation commonly included a part of the splenic artery or vein (Fig. 4b).

## Discussion

The results of this study demonstrate the potential of novel deep learning architectures in postoperative pancreas auto-segmentation on CT images, which has not been studied previously. Our quantitative evaluation reveals the capability of the presented MKNet-family architectures to accurately segment the pancreatic remnant in the postoperative setting over previous approaches, reflected by better HDs and HD95s. Qualitative evaluation demonstrates potential for clinical applicability, making the model valuable for medical education, accelerating postoperative pancreas segmentation, and supporting future research requiring such segmentation, including studies on postoperative complications and detection of local disease recurrence.

Based on quantitative metrics, it was shown that all auto-segmentation models performed substantially worse in the postoperative setting, as compared to the preoperative setting. This became evident after establishing our reference standard, a zero-shot application of TotalSegmentator [9, 29]. Despite its good results in the preoperative setting, its failure to generalize these results in the postoperative setting highlights the magnitude of the domain shift after pancreatic surgery. For the other models, postoperative results remained substantially worse compared with the preoperative results, even after training and fine-tuning on postoperative data. This emphasizes the need to establish dedicated postoperative datasets such as the IMPACT dataset and tailored architectures such as the MKNet-family models. Notably, these results were to be expected considering the smaller pancreas and even wider variation in size, shape and location of the pancreas and surrounding structures after pancreatectomy, demanding substantial model flexibility. For example, mean pancreas size in the postoperative IMPACT dataset was 23,290 mm^3^, while this was 73,510 mm^3^ in the preoperative NIH dataset. Nevertheless, qualitative evaluation by expert abdominal radiologists showed that almost 80% of segmentations by the MSKNet architecture required no to little adjustments. Another interesting finding in this context was that qualitative analysis of the model segmentations seemed to indicate that the model generally performed better after pancreatoduodenectomy than after distal pancreatectomy. Quantitative metrics, however, showed better scores for DSC, HD and HD95 on scans after distal resections, whilst only the NSD was better after head resections. As a higher NSD indicates less under- and overestimation at the border of the pancreatic tissue, e.g., a greater part of the surface voxels is equal to the ground truth, this resonates with the expert analysis. Hence, despite that the quantitative scores can be higher, clinical performance can be lower. This might indicate that achieving a high score on any quantitative metric is not necessarily a truly well-performing model. Considering that our algorithm was capable of segmenting most cases accurately according to expert evaluation, this may provide a better reflection of its true usefulness in clinical practice. Future studies could therefore focus on additional human review when feasible. Furthermore, good qualitative performance of the model seemed related to a sufficiently good DSC and NSD in combination with a very good HD and HD95. This would advocate to focus on optimizing the HD rather than the DSC in future studies, as well as on a combination of quantitative metrics and qualitative metrics, instead of only focusing on the DSC which is often done in medical literature.

DSC scores of MU-Net [16] and PanKNet [17] on preoperative data reported by the original authors could not be reproduced, and a decrease in performance ranging from 7 to 12% was found. For MU-Net [16], this deviation could be caused by a difference in implementation, as a self-implemented version of this model was used. Also, discrepancies could result from a different computation of the DSC. This is particularly relevant in the case of 2D networks, such as in Ma et al. [16] Four-fold division of the data and combining DSCs of the individual slices into the final performance score might substantially alter the findings [41] As identified by Maier-Hein et al. [41], averaging over all slices can result in a substantially higher score than averaging over the slices of one CT scan, with subsequent averaging over all scans. In addition, the DSC can be computed by either using both the foreground and background prediction channels, or using foreground channels only, resulting in different scores. It is generally not mentioned how DSCs are exactly computed, impeding proper comparison between papers. The differences in protocols in the original studies and those used in our study may limit the comparability of reported and achieved performance. However, directly comparing these differences was beyond the scope of the current study.

Visual analysis of the segmentations revealed that the ground truth not always continuously flowed through the slices. This means that the circumference could be equal for several consecutive slices, after which it switched to a rather differently shaped annotation. This is a consequence of the resolution of CT scans as well as the applied preprocessing steps. Slice thickness can be up to four millimeters for some scans, whereas preprocessing using trilinear interpolation enforces the slices to be converted to one millimeter. As mentioned, the masks were reshaped using nearest neighbors to acquire discrete values for ground truth voxels, as suggested by Salanitri et al. [17] In combination with a large slice thickness, however, the nearest neighbor method causes the ground truth to deviate in the intermediate slices. This results in less accurate training and lower measured performance on those slices. As exemplified by Fig. 2, additional tissue in the lower right corner was marked as pancreatic tissue. In the slice below, the ground truth also covered this area. Therefore, it seems that either the ground truth had not been annotated with high enough resolution, implying that the segmentations were more accurate than the ground truth in this particular case, or that rescaling during preprocessing caused the mask to deviate. Although we interpolated the ground truth to a uniform voxel size (1 mm) to facilitate more consistent inter-patient comparisons, we opted not to interpolate the segmentation results back to the original resolution due to concerns about potential artifacts like blurring and aliasing. In this example, addition of attention modules increased accuracy, with the MAKNet and Attention U-Net providing similar segmentations. Nevertheless, MAKNet seemed to follow the ground truth more accurately at the border of the tissue than Attention U-Net. [22] This would indicate better performance of the MAKNet, with more accurate borders likely resulting in a higher NSD. Performance discrepancy may be explained by architectural differences. MAKNet’s multi-scale attention structure promotes better global shape understanding, which may reduce outlier errors and improve HD and HD95 [22]. In contrast, Attention U-Net uses gated skip connections that enhance local detail refinement may improve voxel-level overlap and surface agreement (i.e., DSC and NSD) [16]. These findings may reflect trade-offs and different models may be more applicable for different clinical applications. HD may for example be prioritized when boundary awareness is essential such as in radiotherapy or surgical planning, while DSC or NSD may be preferred for tasks like volumetric analysis. Notably, we hypothesized that skip connections would be redundant for the layer-specific decoders of our MKNet-architectures. However, the MSKNet-architecture performed better than the MKNet-architecture. This suggests that skip connections contribute additional contextual information that aids in better segmentation, especially in the complex postoperative setting.

The model showed systematic errors, such as under-segmentation of atrophic pancreatic tissue and misclassification of peripancreatic tissues like the duodenum or splenic vessels. These errors may impact volumetric accuracy, compromise detection of subtle lesions, or complicate assessment of postoperative complications and radiotherapy planning. Such errors likely arise from low contrast differences between tissues, anatomical variability after surgery, and potentially limited representation of certain patterns in the training data. Although these issues are relevant, the aim of this study was to assess the feasibility and clinical utility of a deep-learning network as a pre-annotation tool to accelerate the initial annotation process and reduce manual workload, and not to replace expert segmentation. Continued architectural refinement to support segmentation workflows in clinical practice is warranted.

The findings of this study need to be interpreted in the light of several limitations. First, the kappa coefficient of 0.23 implies only a fair interobserver agreement. This likely results from the lack of prespecified definitions to classify the outcomes in one of four rather subjective categories. Of note, annotations were made with 3D view, while for clinical evaluation 2D slices were used. Despite the variability, 79% of segmentations were considered to require no or only minor adjustments, and the majority of radiologists were willing to use the model for future pre-annotations. This suggests that although the agreement on specific grading may vary, there is still a shared clinical perception of the model’s usefulness. Second, the output of the visual analysis might question the accuracy of the ground truth used for training of the model. Nevertheless, the majority of the model segmentations were considered clinically useful by expert abdominal radiologists, indicating adequate training and clinical applicability. Furthermore, the observation that the segmentations seemed to outperform the manual annotations for certain cases emphasizes the value of developing computer models to perform complex tasks such as postoperative pancreas segmentation. A follow-up study with expert review of the discrepancies between automated and manual segmentations in such cases could further explore this finding. These feedback loops may furthermore increase the models’ performance. This was, however, beyond the scope of the current study. Third, although all models were fine-tuned on postoperative data using an identical training pipeline, performance in the postoperative setting was generally lower than in the preoperative setting. This emphasizes that preoperative segmentation algorithms are not directly transferable to the postoperative setting, hypothesizing that different aspects may become relevant and guide model performance in this context, thereby emphasizing the relevance of the current study. Last, a well-known issue with regard to the application of deep learning in medical imaging analysis is the limited amount of data available. To increase robustness and accuracy of segmentation models in this context, often a pretraining step is applied, training the model under different conditions, i.e., unsupervised versus supervised, or using different datasets. Learned weights are subsequently transferred to the actual segmentation model to gain prior knowledge about the domain. For this study, we performed pretraining on annotated abdominal CT scans of healthy subjects without pancreatic abnormalities, to increase the performance of the segmentation pipeline. External validation on large, multi-center datasets is essential to confirm the generalizability and robustness of the proposed model.

In conclusion, quantitative and qualitative evaluation of the MKNet-family architecture showed potential to accurately segment the residual pancreas on CT scans after pancreatic resection. This not only advances the state-of-the-art in pancreas segmentation but may also be beneficial for medical application and education, acceleration of data annotation, and be a good ground for future research.

## Supplementary Information

Below is the link to the electronic supplementary material.

Supplementary file1 (DOCX 464 KB)

## Data Availability

The IMPACT Consortium dataset (NCT06055010; https://github.com/IMPACT consortium/IMPACT), comprising 81 annotated postoperative CT scans obtained <4 weeks after pancreatectomy.
