# Supplementary material for: MKNet-family architectures for auto-segmentation of the residual pancreas after pancreatic resection: a deep learning comparative study
Source: Abdom Radiol (NY). 2025 Nov 27;51(7):3492–503. doi: 10.1007/s00261-025-05211-4 (PMC13269288; doi:10.1007/s00261-025-05211-4)
Supplement: Supplementary file 1 — Supplementary file1 (DOCX 464 KB) [file 261_2025_5211_MOESM1_ESM.docx]

**Supplementary Table 1. Performance of current state-of-the-art models for auto-segmentation the pancreas in the preoperative setting using the NIH dataset^30^ as published in the literature**

| **Model** | **DSC ± SD (%)** | **Precision ± SD (%)** | **Sensitivity ± SD (%)** | **HD ± SD (mm)** |
| --- | --- | --- | --- | --- |
| ResDSN C2F^13^ | 84.6 ± 4.9 | NR | NR | NR |
| DQN U-Net^14^ | 86.9 ± 4.9 | NR | NR | NR |
| Two-stage 3D CNN^15^ | 86.0 ± 4.5 | NR | NR | NR |
| MU-Net^16^ | 88.1 ± NR | NR | NR | NR |
| MHSU-Net^16^ | **88.5 ± NR** | NR | NR | NR |
| PanKNet^17^ | 88.0 ± 4.7 | 88.3 ± 5.5 | **88.7 ± 6.0** | NR |
| PanKNet_Light_^17^ | 87.1 ± 4.6 | 86.9 ± 6.5 | 88.5 ± 5.1 | NR |
| Dual-input V-mesh^18^ | 87.4 ± 6.8 | **89.5 ± 5.8** | 87.7 ± 7.9 | 18.4 ± 28.1 |
| CTU-Net^19^ | 86.8 ± 4.1 | 86.2 ± 6.5 | 88.0 ± 6.0 | NR |
| DSC, Dice Similarity Coefficient; SD, standard deviation; NR, not reported  Note: Reported values are drawn from the original studies. Test splits and evaluation setups may differ between models; comparisons are approximate. | | | | |

**
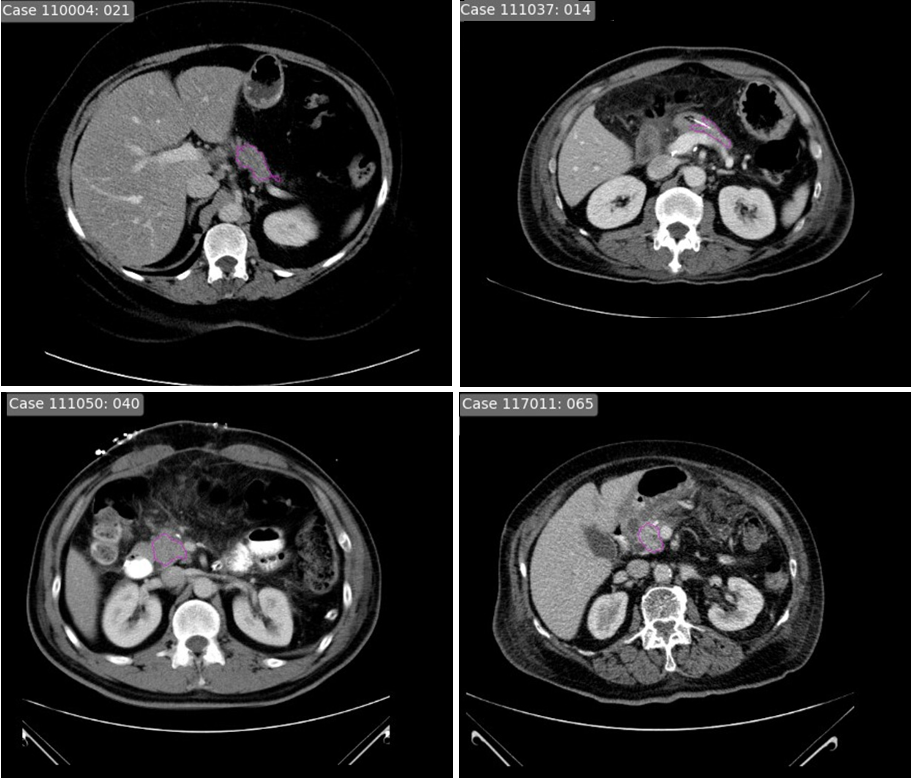
Supplementary Figure 1. Postoperative pancreas segmentations by the MSKNet-architecture with cases 110004 and 111037 after pancreatoduodenectomy and cases 111050 and 117011 after distal pancreatic resection**

**Supplementary Table 2. Sensitivity analysis: quantitative performance of the novel MKNet-family architecture after stratification for resections of the pancreatic head (i.e., pancreatoduodenectomy) or tail (i.e., distal pancreatectomy)**

| **Resection type** | **Pancreatoduodenectomy** | | | | **Distal pancreatectomy** | | | |
| --- | --- | --- | --- | --- | --- | --- | --- | --- |
| **Metric** | **DSC (%), mean ± SD** | **HD (mm) mean ± SD** | **HD95 (mm) mean ± SD** | **NSD (%), mean ± SD** | **DSC (%), mean ± SD** | **HD (mm) mean ± SD** | **HD95 (mm) mean ± SD** | **NSD (%), mean ± SD** |
| MSKNet | 63.5 ± 14.9 | 20.0 ± 13.4 | 13.8 ± 12.7 | 28.2 ± 8.1 | 69.5 ± 7.7 | 15.1 ± 4.9 | 9.7 ± 3.7 | 26.5 ± 5.3 |
| MAKNet | 64.3 ± 15.6 | 18.1 ± 11.7 | 12.2 ± 11.1 | 28.1 ± 8.1 | 69.6 ± 8.0 | 12.8 ± 3.5 | 8.1 ± 3.2 | 25.8 ± 4.8 |
| DSC, Dice Similarity Coefficient; SD, standard deviation; HD, Hausdorff Distance; HD95, 95^th^-percentile Hausdorff Distance; NSD, Normalized Surface Distance | | | | | | | | |
